# Development of a fibromyalgia-specific quality of life instrument: the Fibromyalgia Quality of Life Scale (FM-QoLS)

**DOI:** 10.1007/s00296-025-05895-3

**Published:** 2025-05-16

**Authors:** Ilke Coskun Benlidayi, Ceren Ornek, Aylin Sariyildiz, Yasar Sertdemir

**Affiliations:** 1 Department of Physical Medicine and Rehabilitation, Faculty of Medicine, Cukurova University, Adana, Türkiye; 2 Department of Biostatistics, Faculty of Medicine, Cukurova University, Adana, Türkiye

**Keywords:** Fibromyalgia, Quality of life, Scale, Validation study, Reliability, Surveys and questionnaires

## Abstract

The study aimed to develop a quality of life (QoL) scale specific to patients with fibromyalgia. The scale development involved four steps: (1) Issue collection, (2) Face validity and selection of the most relevant items, (3) Formation of the preliminary scale and pilot testing, and (4) Psychometric evaluation. For psychometric evaluation, the scale was administered to a sample of fibromyalgia patients. Suitability for exploratory factor analysis (EFA) was assessed using the Kaiser–Meyer–Olkin (KMO) test and Bartlett's Test of Sphericity. Reliability was assessed with Cronbach's alpha, Guttman split-half test, and test–retest correlations. Construct validity was determined via EFA using Principal Axis Factoring. The correlation of the developed scale with established measures of QoL, fibromyalgia impact, fatigue, sleep, and mood was tested to assess convergent validity. Floor and ceiling effects were also examined. The initial item list included 25 issues related to QoL in fibromyalgia. Following the assessments and rating of the initial issue list through cognitive interviews, 10 issues were removed. The remaining questions were administered to 60 patients with fibromyalgia. The final version of the Fibromyalgia Quality of Life Scale (FM-QoLS) contained 14 items and two domains: symptomatology-functionality and psychosocial. Moderate-strong correlations between most items indicated that the scale has a homogeneous structure and measures the same construct. The FM-QoLS showed moderate-strong correlations with pain, anxiety, depression, sleep, fatigue, and established measures of QoL. Regarding the test–retest reliability of FM-QoLS items, the gamma values ranged from 0.923 to 0.995. The FM-QoLS is a 14-item QOL scale specific to patients with fibromyalgia. The scale has high validity and reliability, with no floor and ceiling effects. The FM-QoLS could help physicians address the multifaceted QoL challenges in fibromyalgia patients. Future research could refine the scale by exploring its use in different populations.

**Supplementary Information:**

The online version contains supplementary material available at 10.1007/s00296-025-05895-3.

## Introduction

According to the World Health Organization, quality of life (QoL) is defined as “an individual's perception of their position in life in the context of the culture and value systems in which they live and in relation to their goals, expectations, standards and concerns” [1]. It is a multifaceted concept that encompasses the physical, emotional, and social well-being of an individual. Quality of life reflects how individuals feel about their place in life. A systematic review and meta-analysis demonstrated that higher health-related QoL is associated with lower mortality risk [2]. This finding highlights the importance of health status in determining life satisfaction and longevity [2].

Numerous factors can impact QoL, one of which is the presence of a disease. For example, fibromyalgia can affect physical, mental, and social well-being, thus reducing QoL. Fernandez-Feijoo et al. found that patients with fibromyalgia had a poor QoL, with physical pain and vitality being the most affected domains [3]. Depression and anxiety status were identified as the factors most significantly predicting QoL, explaining 49% of the variance. Moreover, polypharmacy and smoking were other predictors of impaired QoL. On the other hand, patients exercising regularly revealed better QoL than those who did not participate in exercise. While these results reflect the dynamic and multi-dimensional nature of QoL, they also underscore the importance of assessing QoL to understand the disease's impact, develop better management strategies, and improve patients’ well-being [3].

Although several standardized tools have been used to evaluate symptom severity, functionality, and disease impact in fibromyalgia (i.e., the Fibromyalgia Impact Questionnaire and its revised version) [4–6], there is no specific standardized questionnaire or scale that directly assesses QoL in fibromyalgia. Instead, in studies, QoL is evaluated by widely-used tools such as the Medical Outcomes Study Short Form (SF)-36 [7], SF-12 [8], Quality of Life Scale (QOLS) [9], EuroQol (EQ-5D) [10], and the Nottingham Health Profile (NHP) [11]. Given the lack in the literature, the current study aimed to develop a fibromyalgia-specific QoL scale.

## Material and methods

The study was approved by the Local Ethics Committee of Cukurova University Faculty of Medicine (Date: 5-April-2024, Number: 143/37). Each patient provided written informed consent prior to participating in the study. Patients (i) aged ≥ 18 years and ii) who were diagnosed with fibromyalgia according to the American College of Rheumatology 2016 Revisions to the 2010/2011 Fibromyalgia Diagnostic Criteria [12] were assessed for inclusion in the study. Exclusion criteria were: (i) physical disability (amputation, hemiplegia, paraplegia, etc.), (ii) severe mental disability (e.g., mental retardation, psychotic disorder), (iii) major organ failure, (iv) any other clinical condition that might have a major impact on QoL, (v) cognitive inability to read or understand, and (vi) illiteracy in the target language.

The study considered recommendations for questionnaire development, as well as guidance for designing, conducting, and reporting survey studies [13, 14]. We also reviewed previous disease-specific QoL scales [15, 16]. It was conducted in four parts: (1) Issue collection, (2) Face validity of the items/selection of most relevant items, (3) Formation of the preliminary scale and pilot test of the scale, and (4) Psychometric evaluation of the scale.

### Issue collection

The goal of this phase is to create a comprehensive list of pertinent QoL concerns covering the topic(s) of interest. Three resources were consulted in the process of creating this list: (i) interviews with fibromyalgia patients, (ii) medical professionals; (iii) literature, including currently available surveys. To guarantee the highest level of content validity in the scale, QoL issues were searched in a patient-centered way [13]. Accordingly, patients with fibromyalgia were asked to think about their disease and identify the factors they believed to have an impact on their everyday life and QoL. In addition, three medical professionals listed the most impactful items (signs, symptoms, and/or social conditions) affecting the QoL of patients with fibromyalgia, based on their clinical judgment. The sample of health care professionals consisted of physicians with at least three years of clinical experience in the field of Physical Medicine and Rehabilitation who work with patients with fibromyalgia [17]. Lastly, a literature search was conducted through PubMed/MEDLINE, Web of Science, and Scopus to identify issues related to QoL in patients with fibromyalgia. The preference was given to the studies published during the last 10 years. Qualitative and quantitative studies about QoL in fibromyalgia were analyzed. Issues that might be related to QoL were extracted [13]. After these steps, the initial issue list was created by compiling the collected items and eliminating duplicates.

### Generating a set of most relevant items/Face validity of the items

Phase 2's objective was to assess the initial issue list through cognitive interviews with health care professionals and patients with fibromyalgia. Face validity of the items was performed in order to select most relevant items [18]. Firstly, the healthcare professionals evaluated the list of QoL issues generated in phase 1 and were asked to identify any missing issues [19]. Then, both the healthcare professionals and patients with fibromyalgia were asked to rate the importance of all issues on a four-point Likert scale, in which 1 represented “not important at all” and 4 indicated “very important”. They were also asked to mark the 10 core issues and to provide feedback on clarity of wording and relevance of each item. Issues were retained if (i) the mean relevance rating was ≥ 2.5 points by patients with fibromyalgia and/or physicians, or (ii) at least 50% of patients with fibromyalgia or physicians considered the issue a priority [17, 19]. The Delphi consultation process resulted in a list of 15 relevant QoL issues [20].

### Formation of the preliminary scale and pilot testing

In this phase, the list of remaining QoL issues was converted into questions. Response categories were as follows: ‘not at all’, ‘a little bit’, ‘moderately’, ‘quite a bit’, and ‘extremely’. For the question asking the level of pain, the responses were: ‘no pain’, ‘a little’, ‘moderate’, ‘quite a bit’, and ‘extreme’. The time frame was set to last two weeks for all items. The development team reviewed and revised the questions for characteristics such as clarity, logical order, response format, and content relevance.

The questions were pilot-tested through cognitive interviews with four patients and four physicians who had not participated in the previous phases of the study. The patients were asked to answer the questions using the think-aloud method. Each participant was requested to provide feedback on the content, format, wording/phrasing, readability, relevance, and feasibility of the questions [19]. Further revisions were made to items, when necessary. The English version of the scale was produced simultaneously with the Turkish version in line with previously published recommendations [21, 22].

### Psychometric evaluation of the scale

Psychometric evaluation of the scale included two steps. First, the theoretical structure of the scale was confirmed through an exploratory factor analysis (EFA). The internal consistency of the scale was evaluated. In the second step, convergent and construct validity of the scale was assessed. Item analysis was performed to identify how well each question contributed to the overall scale. In addition, floor and ceiling effects were checked [23].

To run these analyses, the final version of the scale was administered to a sample of fibromyalgia patients who had not been participated in any of the preliminary phases of the development process. The subject to item ratio was set to 4, which is the frequently recommended approach when performing an EFA [24]. Prior to this, each patient’s sociodemographic data [age, gender, body mass index (BMI), smoking status, alcohol use, annual income, marital status, family and home environment, education level, and employment status] and disease-related variables [disease duration, treatments, Numeric Rating Scale for pain (NRSpain), Widespread Pain Index (WPI), Symptom Severity Scale (SSS)] were recorded.

Along with the FM-QoLS, patients were also asked to complete two widely-used QoL questionnaires/scales including the QOLS and the Nottingham Health Profile (NHP). The QOLS is a psychometric instrument designed to evaluate an individual's perceived QoL across multiple domains. Higher scores reflect a better QoL. The QOLS has been validated in patients with fibromyalgia [9, 25, 26]. The NHP is a 38-item measure designed to assess perceived health-related QoL. It includes 6 domains and the total score for each domain ranges from 0 to 100. Higher scores indicate greater impairment in that domain [27–29].

Disease impact and functionality were assessed by the Revised Fibromyalgia Impact Questionnaire (FIQR). The FIQR includes 3 main domains and a total of 21 items scored on a 0–10 scale. The total FIQR score ranges from 0 to 100. Higher scores indicate worse functional impairment and symptom severity [6, 30]. The Hospital Anxiety and Depression Scale (HADS) was used to evaluate anxiety and depression. It consists of 14 items, divided into two subscales: the anxiety subscale (HADS-A) and the depression subscale (HADS-D). The total score for each subscale ranges from 0 to 21. Higher scores indicate greater symptom severity [31, 32].

Sleep status was assessed using the Jenkins Sleep Scale (JSS). The JSS is a 4-item Likert-type scale, on which the frequency of sleep issues range from 0 (never) to 5 (almost every night). Higher total scores indicate worse sleep quality/status [33, 34]. Fatigue was evaluated using the Fatigue Severity Scale (FSS). The FSS is a nine-item instrument, which addresses the impact of fatigue on daily functioning, its relationship to motivation, physical activity, work, family, and social life. Higher scores indicate more severe fatigue [35, 36].

After two weeks, patients were asked to complete the FM-QoLS once again to evaluate test–retest reliability.

### Statistical analysis

The collected data were analyzed and evaluated using IBM SPSS Statistics for Windows, version 20.0 (IBM Corp., Armonk, NY, USA). The Kaiser–Meyer–Olkin (KMO) test and Bartlett's Test of Sphericity were used to assess the suitability of the data for factor analysis. The reliability of FM-QoLS was assessed using Cronbach’s alpha coefficient and the Guttman split-half test (internal consistency), as well as using test–retest correlations for time invariance (test–retest reliability). Pearson’s and Spearman’s correlation analyses were used for test–retest correlations. Internal consistency was checked using inter-item correlation analysis. Item analysis was performed to evaluate the quality of individual items. Construct validity was investigated with factor analysis. Exploratory Factor Analysis (EFA) was conducted using Principal Axis Factoring to identify the underlying factor structure of the scale. The number of factors was determined based on eigenvalues, the scree plot, and the explained variance. Convergent validity was checked by evaluating whether the FM-QoLS correlated well with other established measures of QoL, including the QOLS and NHP, as well as the FIQR, HADS, FSS, and JSS. Spearman’s correlation analysis was used to test convergent validity. Floor and ceiling effects were checked to ensure that there are no significant proportions of patients scoring at the extreme ends of the scale.

## Results

The initial item list created by collecting items from patients, physicians, and the literature search included 25 issues. Following the assessments and rating of the initial issue list through cognitive interviews, 10 issues were removed, and the list was reduced to 15 issues. After inspection of inter-item correlations, Q15 was found highly correlated with Q12 (correlation coefficient = 0.920). After discussion of their importance, Q15 was removed from the scale.

The FM-QoLS includes 14 items. Response categories range from ‘not at all’ (5 points) to ‘extremely’ (1 point), except for the first question, where responses range from ‘no pain’ (5 points) to ‘extreme’ (1 point). The total FM-QoLS score, which can range from 14 to 70, is obtained by summing the points from each item. Higher scores on the FM-QoLS indicate better QoL in fibromyalgia. Turkish and English versions of the final questionnaire have been provided as Appendix 1 and Appendix 2, respectively.

### Descriptive statistics of the study sample

The psychometric properties of the scale were evaluated using data from 60 patients (57 females, 3 males) with fibromyalgia. The mean age was 45.9 ± 10.6 years and the median disease duration was 60 months. Of the patients, 36.7% were current smokers and 15% were consuming alcohol. The mean years of education was 9.37 ± 4.56 and 41.7% of the patients were employed. In terms of family and home environment, 83.7% of the patients were married, 85% had at least one child, and 6.7% were living alone. Fifteen percent of the patients reported that there are others (e.g., mother-in-law) living in their household besides their immediate family.

### Explanatory factor analysis

Prior to EFA, the KMO test and the Bartlett's Test of Sphericity were used to assess the suitability of the data for factor analysis. The KMO value of 0.921 indicated that the data are highly adequate for factor analysis. The Bartlett’s Test of Sphericity also indicated the appropriateness of the data (approx. chi-square = 785.201, df = 91, p < 0.001).

Table 1 presents the results of EFA using Principal Axis Factoring. The first factor had an eigenvalue of 9.177, explaining 65.55% of the variance. The second factor had an eigenvalue of 1.07, contributing an additional 7.65% and increasing the total explained variance to 73.20%. On the other hand, the third factor’s eigenvalue was < 1. Given the third factor’s minimal explanatory power, Factor 3 was not retained and the findings of EFA supported a two-factor model for FM-QoLS.

**Table 1:** Exploratory Factor Analysis (EFA) was conducted using Principal Axis Factoring

Total Variance Explained							
Factor	Initial Eigenvalues			Extraction Sums of Squared Loadings			Rotation Sums of Squared Loadings^a^
	Total	% of Variance	Cumulative %	Total	% of Variance	Cumulative %	Total
1	9.177	65.55	65.55	8.884	63.457	63.457	8.1
2	1.07	7.645	73.195	0.755	5.392	68.848	7.95
3	0.84	5.997	79.192				

Extraction Method: Principal Axis Factoring

^a^When factors are correlated, sums of squared loadings cannot be added to obtain a total variance

In Table 2, the Pattern Matrix with rotated factor loadings is depicted. Accordingly, Factor 1 had moderate-strong positive loadings on questions Q5-Q11 and Q14. On the other hand, Q1-Q4, Q12, and Q13 were associated with Factor 2. Factor 1 and Factor 2 were categorized as “symptomatology-functionality domain” and “psychosocial domain”, respectively.

**Table 2:** Pattern matrix with rotated factor loadings

	Factor 1	Factor 2
Q10	0.893	
Q9	0.866	
Q6	0.771	
Q8	0.743	
Q11	0.693	
Q7	0.661	
Q14	0.628	
Q5	0.602	
Q4		− 0.994
Q2		− 0.876
Q13		− 0.769
Q1		− 0.755
Q12		− 0.716
Q3		− 0.707

Extraction Method: Principal Axis Factoring

Rotation Method: Oblimin with Kaiser Normalization

Rotation converged in 9 iterations

Loadings below ABS(0.25) were deleted

### Descriptive statistics of the FM-QoLS

The descriptive statistics for FM-QoLS item scores were given in Table 3. Mean scores of the items (Q1-Q14) ranged from 2.017 ± 0.983 to 2.767 ± 1.226. When evaluating the percentage of respondents selecting each option, the proportion of participants indicating a low disease burden with “not at all” responses appeared to be low, in general.

**Table 3:** Descriptive statistics for FM-QoLS item scores

Item	Item score											
	1		2		3		4		5		Mean score	SD
	n	%	n	%	n	%	n	%	n	%		
Q1	23	38.3	18	30.0	14	23.3	5	8.3	0	0.0	2.017	0.983
Q2	22	36.7	19	31.7	14	23.3	5	8.3	0	0.0	2.033	0.974
Q3	21	35.0	17	28.3	13	21.7	9	15.0	0	0.0	2.167	1.076
Q4	24	40.0	15	25.0	17	28.3	3	5.0	1	1.7	2.033	1.025
Q5	13	21.7	13	21.7	17	28.3	13	21.7	4	6.7	2.700	1.225
Q6	23	38.3	7	11.7	15	25.0	13	21.7	2	3.3	2.400	1.291
Q7	23	38.3	12	20.0	13	21.7	11	18.3	1	1.7	2.250	1.202
Q8	7	11.7	18	30.0	20	33.3	13	21.7	2	3.3	2.750	1.035
Q9	16	26.7	13	21.7	17	28.3	11	18.3	3	5.0	2.533	1.214
Q10	14	23.3	16	26.7	15	25.0	12	20.0	3	5.0	2.567	1.198
Q11	9	15.0	21	35.0	10	16.7	15	25.0	5	8.3	2.767	1.226
Q12	16	26.7	20	33.3	19	31.7	3	5.0	2	3.3	2.250	1.019
Q13	15	25.0	23	38.3	15	25.0	5	8.3	2	3.3	2.267	1.039
Q14	17	28.3	22	36.7	16	26.7	5	8.3	0	0.0	2.683	1.142

SD: standard deviation

### Correlation analysis of FM-QoLS items

The results regarding the correlation of FM-QoLS items were provided in Table 4. Moderate-strong correlations across most items indicated that the scale has a homogeneous structure and measures the same construct.

**Table 4:** Correlation analysis of items in FM-QoLS

	Q2	Q3	Q4	Q5	Q6	Q7	Q8	Q9	Q10	Q11	Q12	Q13	Q14
Q1	0.726	0.670	0.723	0.609	0.662	0.585	0.387	0.689	0.683	0.580	0.825	0.825	0.654
Q2	1	0.674	0.814	0.605	0.596	0.529	0.361	0.616	0.623	0.603	0.743	0.728	0.574
Q3		1	0.748	0.540	0.634	0.557	0.327	0.541	0.477	0.556	0.611	0.566	0.526
Q4			1	0.589	0.592	0.544	0.423	0.612	0.564	0.600	0.771	0.771	0.588
Q5				1	0.805	0.616	0.434	0.599	0.626	0.652	0.604	0.609	0.609
Q6					1	0.742	0.456	0.705	0.683	0.713	0.593	0.576	0.639
Q7						1	0.460	0.616	0.653	0.615	0.529	0.543	0.466
Q8							1	0.634	0.635	0.447	0.462	0.488	0.505
Q9								1	0.826	0.700	0.754	0.665	0.784
Q10									1	0.691	0.729	0.652	0.666
Q11										1	0.712	0.621	0.757
Q12											1	0.880	0.768
Q13												1	0.672

Values represent correlation coefficients

P < 0.001 for all correlations

### Convergent validity

The results regarding the convergent validity of FM-QoLS were provided in Table 5. Accordingly, FM-QoLS showed moderate to strong correlations with NHP domains, QoLS, FIQR, NRSpain, HADS-total, HADS-A, HADS-D, JSS, and FSS.

**Table 5:** Convergent validity correlations of FM-QoLS with health-related variables

Spearman’s correlations				
		F1-Total	F2-Total	F1&2-Total
WPI	δ	− 0.646	− 0.603	− 0.643^*^
	p	< 0.001	< 0.001	< 0.001
SSS	δ	− 0.799	− 0.689	− 0.770
	p	< 0.001	< 0.001	< 0.001
NRSpain	δ	− 0.898	− 0.770	− 0.869
	p	< 0.001	< 0.001	< 0.001
FIQR	δ	− 0.873	− 0.865	− 0.917
	p	< 0.001	< 0.001	< 0.001
HADS-total	δ	− 0.681	− 0.848	− 0.815
	p	< 0.001	< 0.001	< 0.001
HADS-D	δ	− 0.584	− 0.722	− 0.697
	p	< 0.001	< 0.001	< 0.001
HADS-A	δ	− 0.702	− 0.866	− 0.836
	p	< 0.001	< 0.001	< 0.001
JSS	δ	− 0.623	− 0.613	− 0.646
	p	< 0.001	< 0.001	< 0.001
FSS	δ	− 0.850	− 0.699	− 0.797
	p	< 0.001	< 0.001	< 0.001
NHP-energy	δ	− 0.722	− 0.647	− 0.707
	p	< 0.001	< 0.001	< 0.001
NHP-pain	δ	− 0.767	− 0.641	− 0.729
	p	< 0.001	< 0.001	< 0.001
NHP-emotional reactions	δ	− 0.704	− 0.850	− 0.823
	p	< 0.001	< 0.001	< 0.001
NHP-sleep	δ	− 0.604	− 0.571	− 0.621
	p	< 0.001	< 0.001	< 0.001
NHP-social isolation	δ	− 0.561	− 0.705	− 0.679
	p	< 0.001	< 0.001	< 0.001
NHP-physical mobility	δ	− 0.751	− 0.726	− 0.770
	p	< 0.001	< 0.001	< 0.001
QOLS	δ	0.736	0.820	0.823
	p	< 0.001	< 0.001	< 0.001

WPI: widespread pain index, SSS: symptom severity scale, NRSpain: numeric rating scale for pain, FIQR: revised fibromyalgia impact questionnaire, HADS: hospital anxiety and depression scale, HADS-D: hospital anxiety and depression scale depression subscale, HADS-A: hospital anxiety and depression scale anxiety subscale, JSS: Jenkins sleep scale, FSS: fatigue severity scale, NHP: Nottingham health profile, QOLS: quality of life scale

### Test–retest reliability

Table 6 presents the test–retest reliability of FM-QoLS items. Accordingly, the gamma values ranged from 0.923 to 0.995. The strong positive associations between test and retest scores suggested excellent test–retest reliability for each item.

**Table 6:** The test–retest reliability of FM-QoLS

	Gamma	*P* value
Q1	0.986	< 0.001
Q2	0.974	< 0.001
Q3	0.987	< 0.001
Q4	0.978	< 0.001
Q5	0.967	< 0.001
Q6	0.981	< 0.001
Q7	0.933	< 0.001
Q8	0.949	< 0.001
Q9	0.923	< 0.001
Q10	0.962	< 0.001
Q11	0.987	< 0.001
Q12	0.969	< 0.001
Q13	0.995	< 0.001
Q14	0.985	< 0.001

The correlations between the test and retest results for the factors are depicted as Supplementary Fig. 1. The mean total score of the FM-QoLS at test and retest were 33.42 ± 12.34 and 34.4 ± 12.61, respectively. The results suggested excellent test–retest reliability for the factors, with Spearman’s rho values of 0.963, 0.975, and 0.966 for Factor 1, Factor 2, and the total, respectively (*p* < 0.001 for all).

### Floor and ceiling effects

The scale has a minimum score of 14 and a maximum score of 70. None of the patients had the lowest and highest scores.

## Discussion

The interest in the assessment of QoL in diseases causing chronic disability has been increasing [15, 16, 37]. The measurement of QoL is quite challenging because it is related to personal perceptions based on various factors such as individuals’ physical and psychological well-being, socioeconomic status, and sociocultural environment. In this regard, surveys/scales/questionnaires are important tools for assessing QoL [38]. These tools not only identify patients’ needs but also provide important information about the effectiveness of the treatment process [39]. There are studies showing that QoL is negatively affected in patients with fibromyalgia. However, these studies have been conducted using the NHP, SF-36, or other tools, which are not specific for fibromaylgia [3, 40, 41]. While general scales evaluate QoL from an inclusive perspective, specific scales can definitely be more effective in making finer distinctions [38]. Filling this gap in the literature, the current study provided a QoL assessment scale specific to patients with fibromyalgia.

It is crucial for QoL assessment tools to demonstrate strong reliability. Reliability refers to the consistency and stability in results. It is essential for accurately capturing and reflecting the true state of an individual's well-being. Given the high Cronbach's alpha value and good test–retest reliability results, FM-QoLS can be regarded as a reliable scale for evaluating QoL in patients with fibromyalgia. Therefore, FM-QoLS can provide stable and consistent responses over time, which enables the scale to be used in studies where repeated measures are required. This is particularly important for studies testing different conditions (e.g., pre- and post-treatment data collection). A scale must also demonstrate robust validity. Validity is critical in ensuring that the tool accurately measures what it is intended to measure. Without strong validity, even the most reliable scale could lead to inaccurate interpretations, misguiding conclusions in studies. Moderate-strong correlations across most items supported the construct validity of FM-QoLS, ensuring that the scale’s items are related while still measuring distinct aspects of QoL in patients with fibromyalgia.

Fibromyalgia is a chronic disease characterized by complex symptoms such as pain, fatigue, sleep problems, and depression [42–44]. Specific surveys that directly assess the multidimensional symptoms of fibromyalgia can help measure the impact of the disease on daily life more precisely. For example, they can provide clear information about which symptoms are more prominent or which areas require more support. The FIQ and its revised version (FIQR) are the most commonly used questionnaires to assess the impact of the disease in patients with fibromyalgia. These tools have also been utilized by researchers aiming to evaluate QoL in fibromyalgia. However, it is important to note that these questionnaires primarily focus on symptoms or functionality. While they provide valuable information about the degree of deterioration and disability caused by the disease, they do not directly assess QoL itself [6, 45, 46]. The concept of QoL is different from deterioration and disability, as it evaluates the effects of the disease based on patients’ own experiences and perceptions rather than clinical data. Therefore, we aimed to develop a scale to understand QoL on a more personal level by directly measuring how much fibromyalgia patients feel the challenges in their social relationships, daily activities, or personal lives. This can enable a better assessment of patients’ perceptions of their QoL. Such a method was also adapted by studies on specific QoL measures of other rheumatic conditions such as ankylosing spondylitis [15].

The current study makes a significant contribution to the literature by developing a disease-specific quality of life (QoL) instrument for fibromyalgia. The questions were tailored to the unique challenges faced by individuals with fibromyalgia. The study provided a patient-centered instrument that reflects the physical, emotional, and social realities of living with fibromyalgia. Furthermore, the development process incorporated input from patients and experts, enhancing both face and content validity.

FM-QoLS can allow for a more comprehensive assessment of the broad impact of fibromyalgia on patients’ daily lives by evaluating various aspects of physical, psychological, and social well-being. For instance, while the FIQ and FIOR typically provide valuable information on functionality, pain, fatigue, sleep, and psychological disorders [4, 6], the FM-QoLS also evaluates additional dimensions of life, such as concentration difficulties, overthinking, social relationships, sexual well-being, and missed opportunities. Inquiries about issues that fibromyalgia patients frequently encounter in their daily lives can help better identify challenges in their daily activities and personal life, as well as barriers in their social relationships [45, 47, 48]. This can lead to more targeted interventions aimed at improving patients’ QoL [49, 50].

In conclusion, FM-QoLS is 14-item QoL scale that is specific to patients with fibromyalgia. The scale has two domains: the symptomatology-functionality domain and the psychosocial domain. It allows for a comprehensive evaluation of fibromyalgia-related deterioration in QoL, with high reliability and validity. Future research could further refine the tool by exploring its use in diverse populations, as well as its role in informing treatment decisions and tracking patient progress over time. Compared to existing generic QoL measures, the FM-QoLS offers a detailed, fibromyalgia-specific perspective, which could help physicians better address the multifaceted challenges faced by patients.

## Supplementary Information

Below is the link to the electronic supplementary material.

Supplementary file 1 (PNG 100 KB)

Supplementary file 2 (DOCX 20 KB)

Supplementary Fig. 1 The correlation of test retest values of factors.

## Data Availability

The data that support the findings of this study are available upon reasonable request from the corresponding author.

## Declarations

### Disclaimer

The authors state that no part of this manuscript, including the text and graphics, are copied or published elsewhere in whole or in part.
