# Supplementary material for: Development of a fibromyalgia-specific quality of life instrument: the Fibromyalgia Quality of Life Scale (FM-QoLS)
Source: Rheumatol Int. 2025 May 16;45(6):142. doi: 10.1007/s00296-025-05895-3 (PMC12084231; doi:10.1007/s00296-025-05895-3)
Supplement: Supplementary file 1 — (PNG 100 KB) [file 296_2025_5895_MOESM1_ESM.docx]

**Fibromyalgia Quality of Life Scale (FM-QoLS)**

INSTRUCTIONS

This questionnaire aims to assess your quality of life in relation to fibromyalgia. Please read the following questions carefully. While thinking about the last 2 weeks, please answer the questions and select only one option for each question.

**1-** **How do you describe your pain level?**

☐No pain ☐A little ☐Moderate ☐Quite a bit ☐Extreme

**2-** **How tired do you feel during the day?**

☐Not at all ☐A little bit ☐Moderately ☐Quite a bit ☐Extremely

**3-** **Do you have sleep problems?**

☐Not at all ☐A little bit ☐Moderately ☐Quite a bit ☐Extremely

**4-** **How tired do you feel when you wake up?**

☐Not at all ☐A little bit ☐Moderately ☐Quite a bit ☐Extremely

**5-** **Do you feel unhappy in general?**

☐Not at all ☐A little bit ☐Moderately ☐Quite a bit ☐Extremely

**6-** **Do you feel anxious in general?**

☐Not at all ☐A little bit ☐Moderately ☐Quite a bit ☐Extremely

**7-** **Do you overthink events or issues?**

☐Not at all ☐A little bit ☐Moderately ☐Quite a bit ☐Extremely

**8-** **Do the attitudes directed toward you by people in your close circle make you unhappy?**

☐Not at all ☐A little bit ☐Moderately ☐Quite a bit ☐Extremely

**9-** **Do you have difficulty with your social activities and relationships (hobbies, relationships with friends and family, etc.) due to your fibromyalgia-related complaints?**

☐Not at all ☐A little bit ☐Moderately ☐Quite a bit ☐Extremely

**10-** **Do you feel like you miss out on the things you want to do and opportunities due to your fibromyalgia-related complaints?**

☐Not at all ☐A little bit ☐Moderately ☐Quite a bit ☐Extremely

**11-** **Do you have difficulty paying attention to a subject or task?**

☐Not at all ☐A little bit ☐Moderately ☐Quite a bit ☐Extremely

**12-** **Do you have difficulty with your daily tasks (housework, work-related tasks, etc.) due to your fibromyalgia-related complaints?**

☐Not at all ☐A little bit ☐Moderately ☐Quite a bit ☐Extremely

**13-** **Do you have difficulty with your daily physical activities (walking, going up and down stairs, etc.) due to your fibromyalgia-related complaints?**

☐Not at all ☐A little bit ☐Moderately ☐Quite a bit ☐Extremely

**14-** **Does fibromyalgia interfere with your private life (including sexual activities)?**

☐Not at all ☐A little bit ☐Moderately ☐Quite a bit ☐Extremely
