# Supplementary material for: Development of a fibromyalgia-specific quality of life instrument: the Fibromyalgia Quality of Life Scale (FM-QoLS)
Source: Rheumatol Int. 2025 May 16;45(6):142. doi: 10.1007/s00296-025-05895-3 (PMC12084231; doi:10.1007/s00296-025-05895-3)
Supplement: Supplementary file 2 — (DOCX 20 KB) [file 296_2025_5895_MOESM2_ESM.docx]

**Fibromiyalji Yaşam Kalitesi Ölçeği (FM-YKÖ)**

YÖNERGE

Bu anket, fibromiyalji hastalığınız ile ilgili yaşam kalitenizi değerlendirmeyi amaçlamaktadır. Lütfen aşağıdaki soruları dikkatlice okuyunuz. Lütfen soruları son 2 haftayı düşünerek cevaplayınız ve her bir soru için yalnızca bir seçeneği işaretleyiniz.

**1-Ağrı düzeyinizi nasıl tanımlarsınız?**

☐Ağrım yok ☐Biraz ☐Orta derecede ☐Fazlaca ☐Aşırı derecede

**2-Gün içinde kendinizi ne kadar yorgun hissediyorsunuz?**

☐Hiç ☐Biraz ☐Orta derecede ☐Fazlaca ☐Aşırı derecede

**3-Uyku sorunlarınız var mı?**

☐Hiç ☐Biraz ☐Orta derecede ☐Fazlaca ☐Aşırı derecede

**4-Uyandığınızda ne kadar yorgun hissediyorsunuz?**

☐Hiç ☐Biraz ☐Orta derecede ☐Fazlaca ☐Aşırı derecede

**5-Genel olarak kendinizi mutsuz hissediyor musunuz?**

☐Hiç ☐Biraz ☐Orta derecede ☐Fazlaca ☐Aşırı derecede

**6-Genel olarak kendinizi endişeli hissediyor musunuz?**

☐Hiç ☐Biraz ☐Orta derecede ☐Fazlaca ☐Aşırı derecede

**7-Olaylar ya da konular üzerinde gereğinden fazla düşünüyor musunuz?**

☐Hiç ☐Biraz ☐Orta derecede ☐Fazlaca ☐Aşırı derecede

**8-Yakın çevrenizdeki kişilerin size karşı tutumları sizi mutsuz ediyor mu?**

☐Hiç ☐Biraz ☐Orta derecede ☐Fazlaca ☐Aşırı derecede

**9-Fibromiyaljiyle ilişkili şikayetlerinizden dolayı sosyal aktivitelerinizde ve ilişkilerinizde (hobiler, arkadaş ve aile ilişkileri vb.) zorluk çekiyor musunuz?**

☐Hiç ☐Biraz ☐Orta derecede ☐Fazlaca ☐Aşırı derecede

**10-Fibromiyaljiyle ilişkili şikayetlerinizden dolayı yapmak istediklerinizden mahrum kaldığınızı ve fırsatları kaçırdığınızı düşünüyor musunuz?**

☐Hiç ☐Biraz ☐Orta derecede ☐Fazlaca ☐Aşırı derecede

**11-Bir konuya ya da işe dikkatinizi vermekte zorlanıyor musunuz?**

☐Hiç ☐Biraz ☐Orta derecede ☐Fazlaca ☐Aşırı derecede

**12-Fibromiyaljiyle ilişkili şikayetlerinizden dolayı günlük işlerinizde (ev işleri, işle ilgili görevler vb.) zorluk çekiyor musunuz?**

☐Hiç ☐Biraz ☐Orta derecede ☐Fazlaca ☐Aşırı derecede

**13-Fibromiyaljiyle ilişkili şikayetlerinizden dolayı günlük bedensel aktivitelerinizde (yürüme, merdiven inip çıkma vb.) zorluk çekiyor musunuz?**

☐Hiç ☐Biraz ☐Orta derecede ☐Fazlaca ☐Aşırı derecede

**14-Fibromiyalji özel hayatınızı (cinsel aktiviteler dahil) etkiliyor mu?**

☐Hiç ☐Biraz ☐Orta derecede ☐Fazlaca ☐Aşırı derecede
