# Supplementary figures and images for: Development of a fibromyalgia-specific quality of life instrument: the Fibromyalgia Quality of Life Scale (FM-QoLS)
Source: Rheumatol Int. 2025 May 16;45(6):142. doi: 10.1007/s00296-025-05895-3 (PMC12084231; doi:10.1007/s00296-025-05895-3)

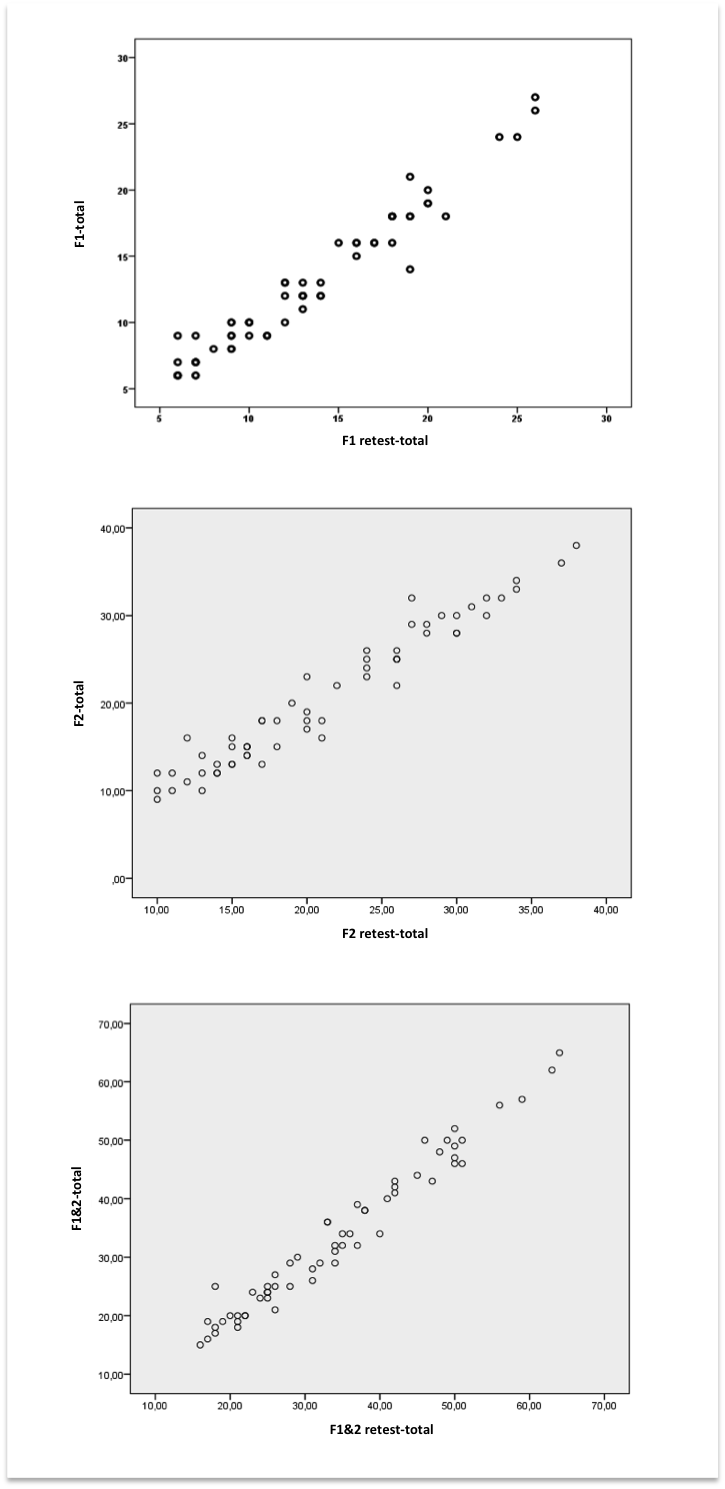

Supplement: Supplementary file 3 — Supplementary Fig. 1 The correlation of test retest values of factors. [file 296_2025_5895_MOESM3_ESM.png]
